# Analysis of chemical constituents and antinociceptive potential of essential oil of *Teucrium Stocksianum* bioss collected from the North West of Pakistan

**DOI:** 10.1186/1472-6882-12-244

**Published:** 2012-12-05

**Authors:** Syed Muhammad Mukarram Shah, Farhat Ullah, Syed Muhammad Hassan Shah, Mohammad Zahoor, Abdul Sadiq

**Affiliations:** 1 Department of Pharmacy, University of Malakand, Chakdara Dir, KPK, Pakistan; 2 Department of Pharmacy, Sarhad University of Science and Information Technology, Peshawar, KPK, Pakistan; 3 Department of Chemistry, University of Malakand, Chakdara Dir, KPK, Pakistan

**Keywords:** *Teucrium stocksianum*, Antinociceptive, Essential oil

## Abstract

**Background:**

Medicinal plants are used for the treatment of different diseases in almost all cultures. Teucrium species grow wildly at different geographical locations around the world. *Teucrium stocksianum* is used in folk medicine for the treatment of diarrhea, cough, jaundice and abdominal pain. Scientific study on *Teucrium stocksianum* shows that it possesses anthelmintic, cytotoxic and antispasmodic activity. The aim of our present study is to identify the chemical composition and antinociceptive potential of the essential oil extracted from *Teucrium stocksianum* bioss.

**Method:**

Essential oil (EO) from the aerial parts of *Teucrium stocksianum* were extracted by hydrodistillation process. The qualitative and quantitative composition of essential oil was determined with Gas chromatography/Mass spectrometer. Antinociceptive activity was determined by acetic acid induced writhing method. Percent inhibition of writhes of the test concentration was determined by comparing it with that of control. Tween-80 emulsion 2.5% (5 ml/kg b.w) was used as a control while Diclofenic sodium 50 mg/kg (b.w) was used as a standard drug.

**Results:**

The chromatogram of the essential oil of *Teucrium stocksianum* shows differences both qualitatively and quantatively from essential oil composition reported in other countries. Hydrodistillation of *Teucrium stocksianum* yielded 0.4% (v/w), pale yellowish oil on dry basis. A total of 26 chemicals were identified by GC-MS accounting for 90.28% of the oil. The major components of essential oil were δ-cadinene (12.92%), α-pinene (10.3%), myrcene (8.64%), β-caryophyllene (8.23%), germacrene D (5.18%) and limonene (2.36%). Essential oil of *Teucrium stocksianum* has shown outstanding antinociceptive activity. It has been observed that increase in percent writhe inhibition (PWI) occurred from 20-80 mg/kg (b.w) and maximum writhe inhibition has been noted at a concentration of 80 mg/kg (b.w), but PWI decreased at 160 mg/kg, which may be due to some toxic effect of higher dose. ED_50_ value for *Teucrium stocksianum* was calculated as 31.5 ± 1.72415 mg/kg (b.w).

**Conclusion:**

Our results indicate that there is a lot of variation in the composition of essential oil of *Teucrium stocksianum boiss,* which may be due to different climatic and experimental conditions. Secondly, the essential oil possesses strong antinociceptive activity and could be used in analgesic preparations especially for topical use.

## Background

Essential oils have been used for therapeutic purposes since ancient times. The Chinese were expert in extraction and use of these oils. Essential oils are produced in specialized ducts called schizogenous ducts in plants. These oils have applications in food industry, fashion and pharmaceutical industry. Such oils have been used as foods preservatives and drugs. Mostly, these are used as topical preparations for analgesics, anti-inflammatory and antimicrobial effects. Nowadays, these are also used as mosquito repellents. All these properties are due to presence of secondary metabolites [1,2].

The genus *Teucrium* belongs to the family Lamiaceae and contains 340 species. Untill now, 04 species have been reported in Pakistan. Medicinal properties of various species of *Teucrium* are reported in literature showing antioxidant, antibacterial, antifungal activities [3-7]. *Teucrium polium* has been reported to possess antispasmodic, antimicrobial, anti-inflammatory, [8], hepatoprotective effect, [9] and analgesic properties [10]. While *Teucrium chamaedrys* has been used as antimalarial, antispasmodic and for gastric pain, kidney disorders, heart diseases etc [11,12].

Lamiaceae family is rich in essential oils. The main components of the essential oil reported from the genus *Teucrium* are alpha pinene, linalool, carophyllene oxide, Germacrene D, beta carophyllene and delta cadinene. These phytochemicals possess antimicrobial, cytotoxic, phospholipase, esterase inhibitory properties [13] and can prove very useful leads for novel drug development.

*Teucrium stocksianum* is a specie found in the North West of Pakistan (Dir, Swat, Malakand, and Hazara). It is a perennial aromatic herb of 10-30 cm height having grayish-white leaves and sessile flowers. It grows in mountains in shades. This plant is used in folk medicine for treating diarrhea, cough, jaundice and abdominal pain [14]. Due to its uses in traditional medicine system, several scientific studies have been conducted to rationalize and establish its therapeutic potential. *Teucrium stocksianum* possesses hepatoprotective and gastric cytoprotective properties [15,16]. Crude saponins isolated from it have shown cytotoxic and anthelminthic effects [17], while antispasmodic activity has also been reported recently. These scientific studies indicate high medicinal potential of *T. stocksianum.* Due to its wild growth in nature and presence of valuable phytochemicals in other species of this genus prompted us to determine chemical composition and therapeutic potential on scientific grounds.

## Methods

### Plant material

The aerial parts of *T. stocksianum* were collected in their full bloom stage from District Dir, in the province of Khyber Pakhtoonkhwa, Pakistan, in May 2012. The plant was dried in shade under suitable condition of temperature in order to avoid any degradation, loss of essential oil and to retain the original colour of the plant. The dried plant was powdered for further use. The plant was identified by Professor Dr Nasrullah, Department of Botany, University of Malakand, Pakistan. A voucher specimen (H.UOM.BG.199) of the whole plant was deposited in the herbarium of the same University.

### Essential oil extraction

The aerial parts of the plant, including inflorescence, small twinges and leaves were cut into small pieces. About 200 g of it was transferred to Clevenger type apparatus and was hydrodistilled for 3-4 h. At the end we got 0.4% (v/w) pale yellowish essential oil based on dried weight of plant. Anhydrous Sodium sulfide (Na_2_SO_4_) was used to dry the essential oil, which was stored at 4°C before analysis. The essential oil was subjected to GC-MS-analysis.

### Chemicals and drugs

All chemicals used in this study were of analytical grade purchased from Merck, Pakistan. Diclofenic sodium was obtained from Sigma.

### Statistics and calculations

One-way analysis of variance (ANOVA) and Tukey’s multiple comparison test was applied for the comparison among various groups. Differences with *P ≤* 0.05 and lower between groups were considered significant.

### Gas chromatography–mass spectrometry

The components of the EO were analyzed using GC-MSQP 2010 (Tokyo, Japan), with an auto-injector (AOC-20i) and auto-sampler (AOC-20s). Sample was eluted with Helium gas. Components were separated with capillary column (DB-5MS Prepared by Agilent Technology USA) having 30 m length, 0.250 mm internal diameter and 0.25 micro meter thickness. Electron impact ionization mode with energy 70 ev, ion source temperature 250°C, interface temperature 240°C with 80 KPa pressure, 1.8 min solvent cut time. Injector temperature was 250°C and operated in split mode with 1 ml/min. The column was programmed at a temperature of 50°C for 1 min initially and then changed to 150°C at the rate of 15°C/min and kept constant 15 min. The column temperature was increased to 280°C at a rate of 2.5°C per min and was maintained for 3 min. Mass spectra were acquired in the range of 40 to 650 m/z*.* A series of normal alkanes was also injected under same analytical conditions with that of the essential oil for the calculation of Retention Indices. Components of the essential oil were identified by comparing the mass spectra obtained with those of standard mass spectra from the NIST library (NIST 05). Relative concentration of the components was calculated from the peak areas of the total ion chromatograms.

### Animals

Swiss Albino mice of either sex, weighing between 20-30 g of approximately same age were used for the present study. Animals were kept under controlled laboratory conditions on 10/14 h light and dark period with free access to laboratory diet and water. All animals were fasted for 24 h before the test. Animal’s studies were performed according to the Scientific Procedures Issue-1 of Animal Bylaws-2008 approved by the legal bodies of the University of Malakand Khyber Pakhtoonkhwa, Pakistan. The ethical committee of the department of pharmacy granted approval for conducting this study under the said protocols (Procedures Issue-1 of Animal Bylaws-2008).

### Antinociceptive activity

*Acetic acid induced writhing test*; Swiss Albino mice of both sexes were divided into five groups, each including six animals. 2.5% Tween-80 solution (10 ml/kg) was administered intraperitoneally to control group. Test groups were treated with an emulsion of *Teucrium stocksianum* essential oil of Tween-80 (2.5% v/v, water as a vehicle), at a dose of 20-160 mg/kg and standard group received intraperitoneal injection of 50 mg/kg dose of Diclofenic sodium. After 30 min, 0.6% acetic acid (15 ml/kg) was injected through the same route. Total writhes produced in each mouse were noted for 20 min immediately after injection of acetic acid. Antinociception was determined by reduction of writhing numbers by comparing the number of writhes produced in the control group treated with Tween-80 and to that of test groups treated with *Teucrium stocksianum* essential oil doses of 20-160 mg/kg [18].

## Results and discussion

In this study, hydrodistillation of the aerial parts of *T. stocksianum* gave pale yellow coloured essential oil having a pleasant aromatic smell. Yield of the oil was about 0.4 (v/w) on a dry weight basis. A total of 26 chemicals were identified by GC-MS accounting for 90.28% of the oil, given in the Table 1. Constituents of essential oils vary with geographic location, collection time and parts of plants used for oil extraction. Bagci et al. have reported 36 chemicals from *T. chamaedrys* collected from Turkey [19] while Morteza-Semmani K et al. reported 49 chemical constituents from the essential oil of same specie from Iran [20]. Yousuf et al., have reported differences in the number of chemicals and relative concentration of chemicals in the same specie from same geographic location, but collected in different years [21]. Sesquiterpenes (59%) were found more than monoterpenes (40.97%) in this study. Other studies done on *Tecucrium* genus have also recorded more sesquiterpenes than the monoterpenes in their essential oils [19,22]. Relative concentration of different major components observed in our study are, δ-cadinene (12.92%), α-pinene (10.3%), myrcene (8.64%), β-caryophyllene (8.23%), germacrene D (5.18%), limonene (2.36%), elemol (2.13%), γ-cadinene (1.86%). While comparing relative concentration of different components of the essential oil from same specie, we recorded δ-cadinene to be 12.92% while Yousuf et al. have recorded 13.8% δ-cadinene from UAE [21], which is almost the same value. It is to be noted that major chemical constituent of our study is δ-cadinene in contrast to Yousuf et al. and Jaimand et al., who have recorded α-cadinol (14.6%) and camphene (20.6%) as the major constituents in the United Arab Emirates (UAE) and Iran respectively [21,23]. Literature shows great variation in the concentration of α-pinene in the Tecucrium genus. In our study, α-pinene was the second most abundant component while Moghtader et al. from Iran has reported the same component to be 12.52% as the major one in their study on the essential oil of *Teucrium stocksianum*[24]. In contrast, Nasser et al. from Yemen and Bagci et al. from Turkey have reported α-pinene, 0.96% and 0.2% respectively [19,25]. It is worth noting that Jaimand et al. from Iran and Yousuf et al. from the United Arab Emirate (UAE) haven’t found this component in their work [21,23]. Germacrene D, a common sesquiterpene, has been found by researchers from the genus *Teucrium* genus from different countries like Iran (10.2%) [26], Jordan (4.3%) [27], Italy (18%) [28] and Portugal (21.6%) [29]. This compound may be a precursor for the biosynthesis of various sesquiterpenes like cadinenes and selinenes [30] and is insecticidal against mosquitoes [31]. We found it to be 5.18% in our study.

**Table 1:** **Percentage composition of the essential oil from the aerial part of*Teucrium stocksianum*Bioss**

**Compound No.**	**Compound Name**	**Retention Indices**	**Percentage**
1	α Pinene	943	10.3
2	Sabinene	971	2.6
3	β Pinene	986	2.82
4	Myrcene	1008	8.64
5	α Terpinene	1020	1.21
6	Cymene	1030	0.85
7	Limonene	1041	2.36
8	Terpineol	1146	0.49
9	Linalool	1108	1.75
10	Myrienal	1210	2.75
11	Bornyl acetate	1292	1.58
12	β Myrcene	1303	1.64
13	6 Elemene	1345	2.95
14	α Cubebene	1359	1.72
15	α Copaene	1385	1.51
16	β Cubebene	1404	0.53
17	β Caryophyllene	1427	8.23
18	α Guaiene	1445	2.53
19	α Humulene	1462	2.81
20	Seychellene	1479	6.72
21	Germacrene D	1488	6.18
22	ϒ Cadinene	1519	2.86
23	6 Cadinene	1535	12.92
24	Spathulenol	1558	1.35
25	Elemol	1557	2.13
26	Caryophyllene oxide	1590	0.85
	Total		90.28

*RI* = Retention indices.

Scientific studies are conducted globally to evaluate antinociceptive efficacy of essential oils. The data from such studies shows that essential oils of various plants containing chemical constituents exert good antinociceptive effects through various mechanisms [32]. Although medicinal plants are often tested for their therapeutic effect as a whole in experiments, studies are available in which single chemical constituents have been tested. Him et al. and Ozbek et al. have evaluated a positive analgesic effect of alpha pinene in their study [33,34]. Similarly analgesic activity of myrcene [35], limonene [36], linalool [37] and caryophyllene oxide [38] have been observed.

The chemical method i.e, acetic acid induced writhing protocol is most commonly used for evaluating antinociceptive activity of medicinal plants. Prostaglandins, initially PGE2 and then PGF2α and free arachidonic acid are released from tissue phospholipids and consequently their levels in the peritoneal fluids increase due to intraperitoneal administration of the irritant, acetic acid. This results in localized inflammatory response and pain sensation due to increase in capillary permeability. Substances which counteract this phenomenon, exert antinociceptive effect and reduce pain sensation [39]. A plethora of studies is available which conclude antinociceptive activity of essentials oils of medicinal plants [40-42]. Various species of the genus *Teucrium* possess antinociceptive activity [10,25,43]. Abdollahi et al. in 2004 conducted a study in Iran and concluded that essential oil was the main contributor in antinociceptive activity [10]. In our study, the essential oil of *Teucrium stocksianum* significantly inhibited the number of abdominal constrictions induced by acetic acid (Table 2). The essential oil at a dose of 20 mg/Kg, decreased writhing by 47% (*p* value <0.001), at 40 mg/Kg 83% (*p* value <0.001), at 80 mg/Kg 93% (*p* value <0.001) and at 160 mg/Kg 69% (*p* value <0.001) writhing were inhibited. The positive control used Diclofenic sodium at 50 mg/Kg inhibited 70% writhing. Thus, the essential oil in our study had strong activity than the positive control at doses above 40 mg/Kg. Essential oil of *Teucrium stocksianum* is a combination of various components as shown in Table 1, in which alpha pinene, myrcene, limonene, linalool and caryophylene oxide are present in reasonable concentrations and all these components possess antinociceptive effect. High potency of essential oil of *Teucrium stocksianum* as compared to Diclofenic sodium may be due to the synergistic effect of the various components present in the essential oil.

**Table 2:** **Antinociceptive effect of essential oil of*Teucrium stocksianum*bioss on abdominal writhing in mice indused acetic acid abdominal injection**

**Dose (mg/kg)**	**N**	**Number of wriths in 20 min (mean ± S.D)**	**% Inhibition**
Control (10 ml/kg) Essential oil	6	58 ± 5.600	
20	6	30.333 ± 2.42212	47.701**
40	6	9.6667 ± 2.16025	83.333**
80	6	3.5000 ± 1.64317	93.965**
160	6	17.5000 ± 3.50714	69.827**
Diclofenic sodium	6	17.3333 ± 3.55903	70.115**

*N*: Number of animal per group. *D.Sod*: Diclofenic Sodium. Percent inhibition was calculated in comparison to control. *F =* 190.486*, d.f. =* 35. ED_50_ for analgesic effect of essential oil was 31.5 ± 1.72415 mg/kg (b.w). The significance showed by ** P ≤ 0.05.

The results of antinociceptive effect of our study are similar to that of essential oil extracted from *Teucrium polium* with respect to antinociceptive potential*. T stocksianum* caused 93% writh inhibition at a dose of 80 mg/kg, while *T polium* produced similar effect (90.22%) at a dose of 75 mg/kg (Abdollahi, Karimpour et al. 2003;). This resemblance may be due to qualitative and quantitative similarities in the chemical composition of the essential oil of the two species. Furthermore *Teucrium stocksianum* is abundantly available in the North West of Pakistan and it grows wildly. It is commonly used in the folk medicinal system of this region. Our study has explored a new source of a very potent and economic analgesic. Such type of studies in which indigenous resources are used would not only provide effective and economical remedies but would also help in poverty reduction of the region.

## Conclusion

Our result pertaining to the composition of essential oil has shown that the composition of the essential oil of the same specie collected from different geographical locations and in different seasons are not the same. Essential oil of *Teucrium stocksianum* has been evaluated by Moghtader et al. from Iran, Nasser et al. from Yeman, Bagic et al from Turky, Yousuf et al. from United Arab emirates (UAE) et al. The results of all these researcher shows a lot of qualitative and quantitative variation in composition (19-21).

Furthermore, essential oil of *Teucrium stocksianum* possesses a strong antinociceptive potential, which needs further scientific investigation for the rational use as a topical analgesic.

## Competing interests

The authors declare that they have no competing interests.

## Authors’ contributions

SMMS carried the practical work, SHS and MZ conceived the idea and did literature survey, FU, SMMS and AS drafted the manuscript. All authors read and approved the final manuscript.

## Pre-publication history

The pre-publication history for this paper can be accessed here:

http://www.biomedcentral.com/1472-6882/12/244/prepub
